# Enhancing the oral and topical insecticidal efficacy of a commercialized spider venom peptide biopesticide via fusion to the carrier snowdrop lectin (*Galanthus nivalis* agglutinin)

**DOI:** 10.1002/ps.7198

**Published:** 2022-10-10

**Authors:** Nur Afiqah Sukiran, Prashant Pyati, Caitlin E Willis, Adrian P Brown, Jennifer J Readshaw, Elaine C Fitches

**Affiliations:** ^1^ School of Biosciences University of Durham Durham UK; ^2^ Ajeet Seeds Pvt. Ltd. Plant Biotechnology Research Centre Aurangabad India; ^3^ Biointeractions & Crop Protection Department Rothamstead Research Harpenden UK

**Keywords:** fusion protein, hexatoxin, snowdrop lectin, aphids

## Abstract

**BACKGROUND:**

Spear®‐T sold as a contact foliar spray for the control of glasshouse pests such as aphids, thrips, spider mites and whiteflies, contains the recombinant spider venom peptide GS‐ω/κ‐HxTx‐Hv1h (named as GS‐ω/κ‐HxTx‐Hv1a by Vestaron) as the active ingredient. Here we investigate whether fusion of the peptide to snowdrop lectin, (*Galanthus nivalis* agglutinin; GNA) enhances the efficacy of this venom peptide towards aphid pests.

**Results:**

Recombinant GS‐ω/κ‐HxTx‐Hv1h (HxTx‐Hv1h) and an HxTx‐Hv1h/GNA fusion protein were produced using the yeast *Pichia pastoris*. Purified proteins showed comparable toxicity when injected into lepidopteran (*Mamestra brassicae*) larvae, but significant differences in oral and contact activity towards aphids. HxTx‐Hv1h had comparable acute oral toxicity to pea (*Acyrthosiphon pisum*) and peach potato (*Myzus persicae*) aphids with respective Day (2) median lethal concentration (LC_50_) values of 111 and 108 μm derived from diet assays. The fusion protein also showed comparable oral toxicity to both species but D2 LC_50_ values were >3‐fold lower (35 and 33 μm for pea and peach potato aphids, respectively) as compared to HxTx‐Hv1h. Topically applied toxin and fusion protein, but not GNA, caused significant reductions in pea aphid survival. Contact effects on mortality were significantly greater for aphids exposed to fusion protein as compared to toxin alone. Whole aphid fluorescence microscopy and immunoblotting suggest that improved efficacy is due to enhanced persistence of HxTx‐Hv1h when fused to GNA following internalisation of ingested or topically applied proteins.

**Conclusions:**

This is the first study to report on the insecticidal activity of HxTx‐Hv1h towards aphids and results suggest that a fusion protein‐based approach offers opportunities to significantly enhance oral and contact efficacy of naturally derived toxins, such as HxTx‐Hv1h, towards crop pests. © 2022 The Authors. *Pest Management Science* published by John Wiley & Sons Ltd on behalf of Society of Chemical Industry.

## Graphical abstract

We have produced a recombinant spider venom derived peptide (HxTx‐Hv1h) and a fusion protein linking HxTx‐Hv1h to snowdrop lectin (GNA) and subsequently shown that the fusion protein has greater oral and contact insecticidal activity as compared to HxTx‐Hv1h towards two pest species of aphids.

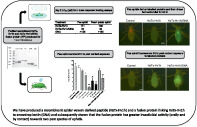

## 1 INTRODUCTION

As venomics research progresses, spider venoms are increasingly being recognized as a rich and valuable source of neurotoxins providing a growing pool of candidates with potential for development as novel biopesticides. Many spider venom peptides are short (2.5–5 kDa) and disulfide‐rich, and the most characterised are those containing an evolutionarily conserved inhibitor cystine knot (ICK) motif.^1^ The ICK motif, defined as an antiparallel ß‐sheet stabilised by a cystine knot,^2^ provides high levels of chemical and thermal stability as well as resistance to proteolytic degradation in the insect gut and haemolymph.^3^, ^4^, ^5^ These properties are important for commercial production and viability as biopesticides. Indeed, the registration of Spear®‐T by Vestaron in 2014, which contains the spider venom neurotoxic peptide GS‐ω/κ‐HxTx‐Hv1h (hereafter referred to as HxTx‐Hv1h) as the active ingredient, provides definitive evidence of the potential for the commercialisation of venom peptide‐based biopesticides.

HxTx‐Hv1h is a member of the ω‐hexatoxin‐1 (HxTx) family which were among the first peptides isolated from the venom of Australian funnel‐web spider, *Hadronyche versuta*.^6^, ^7^, ^8^ Well‐studied members of subgroups of the hexatoxin superfamily, thought to have evolved from a common ancestral gene, also include ω‐hexatoxin‐Hv1a and κ‐hexatoxin‐Hv1c. All three toxins have been shown to be potently insecticidal by injection into a range of insects, but harmless to mammals, and surprisingly nontoxic to honeybees.^9^ ω‐hexatoxin‐Hv1a initially was shown to target insect voltage gated calcium channels (Ca_v_), whereas κ‐hexatoxin‐Hv1c targets Ca‐activated potassium channels (K_Ca_).^8^, ^10^, ^11^, ^12^ Whilst structurally similar, the mature amino acid (aa) sequences of the three toxins are diverse. HxTx‐Hv1h has been designated as a hybrid toxin as it contains critical aa residues that are present in either ω‐hexatoxin‐Hv1a or κ‐hexatoxin‐Hv1c.^11^, ^13^ Early work by Sollod (2006) suggested that this hybrid toxin, which was the most efficacious of the three toxins when injected into houseflies (*Musca domestica*), is the most potent due to synergistic disruption of both Ca_v_ and K_Ca_. More recently, neuronal membrane binding studies have shown all three toxins to have more potent effects via their action as positive allosteric modulators of insect nicotinic acetylcholine receptors (nAChR).^13^ Acetylcholine receptors mediate the actions of acetylcholine, the primary excitatory neurotransmitter in the insect central nervous system (CNS). Resistance development to chemical pesticides that target sites within the insect nervous system is widespread and can occur via mutation (e.g. aphid resistance to pyrethroids is achieved via mutations in voltage‐gated sodium channels).^14^ Thus, consideration of the different target sites of action of hexatoxin peptides is key to understanding their potential development as resilient biopesticides. The lack of toxicity of hexatoxins towards honeybees may be attributable to diversity in insect nAChR receptor subunits and the presence of multiple nAChR subtypes; notably, the honeybee has been shown to possess a larger number of nAChR subunits as compared to the fruit fly *Drosophila melanogaster* and mosquito *Anopheles gambiae*.^15^

Many venom ICK peptides are highly potent by injection but typically much less effective when ingested.^16^ Neurotoxic peptides must access the CNS or peripheral nervous system (PNS) to reach their target site(s) of action, and failure of ingested peptides to cross the gut epithelium is thought to be the major barrier to achieving oral efficacy. We have demonstrated previously that fusion of several different venom neurotoxins, including ω‐hexatoxin‐Hv1a, to the snowdrop lectin *Galanthus nivalis* agglutinin (GNA) carrier protein dramatically enhances oral insecticidal activity.^17^, ^18^, ^19^, ^20^, ^21^, ^22^ GNA is able to transport attached peptides across the insect gut allowing delivery to the circulatory system. Likewise, Hv1a when fused to a luteovirus coat protein, which crosses from the aphid gut lumen to the haemocoel has been shown to significantly enhance the oral efficacy of Hv1a towards aphids.^23^ Furthermore, GNA has been shown to bind to the central nerve chord of lepidopteran larvae and, therefore, also may mediate delivery of attached toxins to sites of action within the CNS.^19^

The commercial HxTx‐Hv1h product is sold as a contact foliar spray pesticide for the control of glasshouse pests such as aphids, thrips, whitefly and two‐spotted spider mite (see www. vestaron.com for more information). Nontoxicity towards biological control agents (rove beetle *Dalotia coriaria* and flower bug *Orius insidiosus*) commercially used in glasshouses has been reported.^24^ However, published data regarding the efficacy of the commercial HxTx‐Hv1h product against target pests is limited. Low topical and/or residual efficacy of HxTx‐Hv1h towards adult fruit fly (*Drosophila suzukii*) has been reported although efficacy was significantly enhanced when the peptide was combined with adjuvants presumed to facilitate paracellular transport across the insect cuticle.^25^ The commercial product was reported to be ineffective against *M. domestica*) when applied at a maximum rate of 5000 ppm as a fog/mist either alone or in combination with the adjuvant Silwett L‐77.^26^

This paper reports on the production and efficacy evaluation of recombinant HxTx‐Hv1h and a HxTx‐Hv1h/GNA fusion protein. We tested the hypotheses that fusion of the HxTx‐Hv1h toxin to GNA would enhance the oral and contact efficacy of the venom neurotoxin. Oral toxicity of both proteins towards aphids; pea aphid, *Acyrthosiphon pisum* and peach potato aphid, *Myzus persicae* and contact activity towards pea aphids is reported. Overall, our results demonstrate that fusion of GS‐ω/κ‐HxTx‐Hv1h to GNA does indeed enhance both oral and contact toxin efficacy towards aphids.

## 2 MATERIALS AND METHODS

### 2.1 Materials

A *Pichia pastoris* codon‐optimised nucleotide sequence encoding ω/κ‐hexatoxin‐Hv1h (accession no. S0F209; residues 38–76) hereafter referred to as HxTx‐Hv1h, and cloning primers were purchased from Integrated DNA Technologies (IDT, Coralville, IA, USA). Restriction endonucleases were supplied by Thermo Fisher Scientific (Waltham, MA, USA) or New England BioLabs (Ipswich, MA, USA). Electrophoresed DNA fragments were purified from excised gel slices using a Qiagen gel extraction kit. Plasmid DNA was prepared using Promega Wizard miniprep kits. T4 ligase kit was supplied by Promega. Phusion polymerase was from New England Biolabs. *P. pastoris* (SMD1168H strain), the expression vector pGAPZαB and Easy comp *Pichia* transformation kit were from Invitrogen (Thermo Fisher Scientific).

Anti‐GNA antibodies were prepared by Genosys Biotechnologies (Cambridge, UK). Monoclonal 6x‐His Tag Antibodies were from Thermo Fisher Scientific. Secondary IgG horseradish peroxidase antibodies were from BioRad (Hercules, CA, USA). Chemicals for chemiluminescence and buffer salts were supplied by Sigma Aldrich (St Louis, MO, USA).

### 2.2 Assembly of HxTx‐Hv1h and HxTx‐Hv1h/GNA fusion protein expression constructs

**Figure 1:**
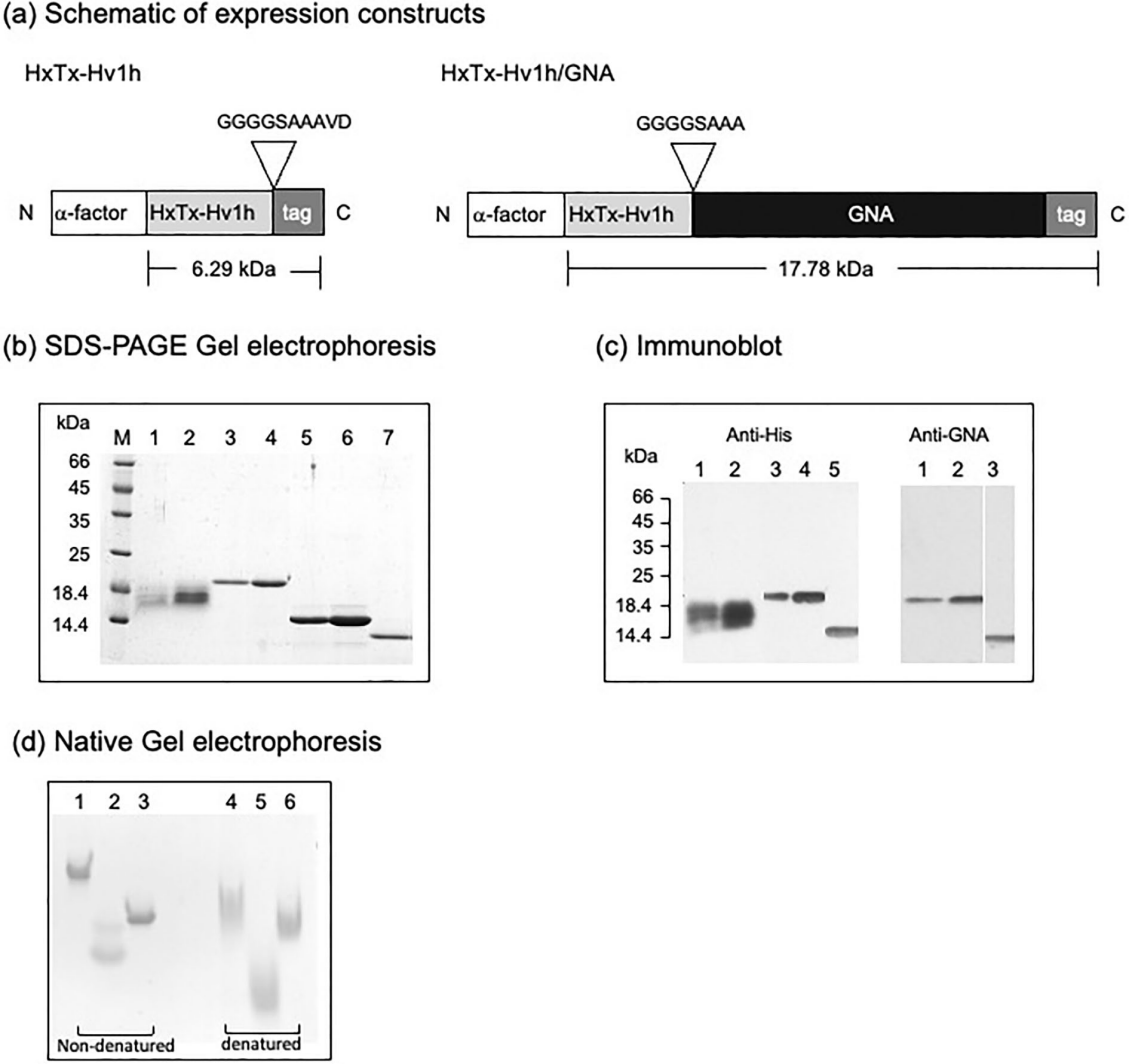
(a) Schematic of constructs encoding recombinant HxTx‐Hv1h and HxTx‐Hv1h/GNA produced in the yeast *P. pastoris* showing predicted molecular masses; tag denotes the presence of a six‐residue histidine sequence that allows protein purification by nickel affinity chromatography and detection by Western blotting. (b) Separation of purified proteins by SDS‐PAGE gel stained for total protein: lanes 1 and 2 are HxTx‐Hv1h (*c*. 1 and 3 μg, respectively), lanes 3 and 4 are HxTx‐Hv1h/GNA (*c*. 2 and 3 μg, respectively), lanes 5 and 6 are recombinant GNA (*c*. 3 and 5 μg, respectively) and lane 7 is Sigma GNA standard (2 μg). (c) Western analysis of recombinant proteins using anti‐His [lanes 1–5 are as for (b); loading *c*. 50 and 100 ng for HxTx‐Hv1h and HxTx‐Hv1h/GNA and 50 ng for GNA] and anti‐GNA (lanes 1 and 2 are, respectively, 50 and 100 ng HxTx‐Hv1h/GNA, lane 3 is Sigma GNA standard 50 ng) antibodies. Location of mass markers run on the same gel are depicted. (d) Separation of nondenatured and denatured proteins by native PAGE, gel‐stained for total protein (15 μg loaded in each lane). Lanes 1 and 4 are HxTx‐Hv1h/GNA, lanes 2 and 5 are HxTx‐Hv1h, and lanes 3 and 6 are recombinant GNA.

The HxTx‐Hv1h coding sequence was amplified by PCR using primers containing *Pst*I and *Sal*I restriction sites. Following gel purification, the PCR product was digested (*Pst*I and *Sal*I) and ligated into similarly cut vector, pGAPZαB DNA. To generate a fusion protein construct where HxTx‐Hv1h is linked to the N‐terminus of GNA, the toxin coding sequence [Fig. 1(a)] was amplified by PCR (using primers containing *Pst*I and *Not*I restriction sites), gel‐purified, restricted and ligated into a previously generated pGAPZαB construct that contained a GNA coding sequence. Plasmids were cloned into electrocompetent *Escherichia coli* (DH5α) cells and DNA coding sequences were verified by ‘in house’ DNA sequencing.

### 2.3 Yeast transformation, expression and purification of recombinant proteins

Plasmid DNAs from sequence‐verified clones were linearised with *Avr*II and transformed into chemically competent *P. pastoris* cells according to the manufacturer's instructions. Transformants were selected on medium containing 100 μg mL^−1^ zeocin. Clones expressing HxTx‐Hv1h or HxTx‐Hv1h/GNA were selected for production by bench‐top fermentation by Western analysis (using anti‐His or anti‐GNA antibodies) of supernatants from 10 mL cultures grown at 30 **°**C for 2–3 days in YPG medium [1% (w/v) yeast extract, 2% (w/v) peptone, 4% (v/v) glycerol, 100 μg mL^−1^ zeocin] (results not shown).

For protein production, *P. pastoris* cells expressing HxTx‐Hv1h or HxTx‐Hv1h/GNA or GNA were grown in a bench top fermenter (ez‐control Applikon 7.5 L vessel) as described previously.^18^ Following fermentation, proteins were separated from cells by centrifugation (20 min at 7000 × *g*, 4 **°**C) and purified via nickel affinity chromatography as described previously.^26^ Pooled fractions containing purified proteins were dialysed against distilled water and lyophilised. Protein contents in lyophilised samples were determined from SDS‐PAGE gels stained for total proteins with Coomassie blue. Known quantities of lyophilised samples were re‐suspended in 50 mm sodium phosphate buffer pH 7.4 (SPB) to generate stock solutions from which dilutions of known amounts of powder were loaded on gels; six samples of different known powder quantities were loaded alongside six GNA standards. Quantitation was based on densitometric analysis of protein bands, which were compared to GNA (Sigma Aldrich) standards by visual inspection, and ibright analysis of gel images scanned using a commercial flat‐bed scanner.

### 2.4 Electrophoresis, Western blotting and fluorescein conjugation

SDS‐PAGE electrophoresis, Western blotting and fluorescein isothiocyanate (FITC) labelling of proteins was carried out as described previously.^19^ Proteins also were separated by native PAGE electrophoresis [acrylamide 2.1% (v/v) stacking and 11.3% (v/v) resolving gel] in the absence of SDS. Nondenatured protein samples were prepared by the addition of 5X native PAGE buffer [12.5% (v/v) 0.5 m Tris HCl pH 6.8, 25% (v/v) glycerol, 62.5% (v/v) Milli‐Q water] to aliquots of protein stock solutions (2 mg mL^−1^ in distilled water). Denatured samples were prepared by addition of 5X native PAGE buffer with the addition of 50 mm Tris (2‐carboxyethyl) phosphine hydrochloride (TCEP) and boiled for 10 min at 100 **°**C. Samples were cooled to room temperature and centrifuged briefly before 10 μL of 8 m urea was added to solubilise the proteins. The gels were run in an ATTO‐AE6450 gel tank apparatus containing 1X reservoir buffer (25 mm tris, 192 mm glycine) at 70 V for 4 h. Gels were stained for total protein with Coomassie Brilliant Blue.

### 2.5 Recombinant protein characterization

Recombinant HxTx‐Hv1h and HxTx‐Hv1h/GNA were separated by SDS‐PAGE and excised bands from gels stained with Coomassie Blue were analysed by liquid chromatography–mass spectrometry (LC–MS). Proteins in excised bands were digested with chymotrypsin and/or trypsin and LC–MS analysis was performed with a Sciex TripleTOF 6600 mass spectrometer coupled to an ekspertTM nanoLC 425 with low micro‐gradient flow module (Eksigent Technologies, Redwood City, CA, USA) via a DuoSpray source (Sciex, Framingham, MA, USA) as described previously.^21^

The glycosylation status of purified proteins was investigated by Periodic Acid Schiff staining. Proteins transferred to nitrocellulose membranes were stained with Ponceau S (to visualise molecular mass proteins), de‐stained and washed in distilled water. Membranes were incubated in Solution A [1.0% (v/v) Periodic acid, 3.0% (v/v)] for 30 min, washed in distilled water, and further washed twice (5 min each) in Solution B [0.1% (w/v) sodium metabisulphite] and then incubated for 15 min in Schiff's reagent (Sigma Aldrich). Finally, membranes were washed twice in the dark with Solution B, air‐dried for 1 h and imaged on a scanner.

### 2.6 Insect rearing

*Mamestra brassicae* originally obtained from cultures held at Fera Science Ltd were reared at the University of Durham continuously on artificial diet^17^ at 22–25 **°**C with 65% RH under 16 h: 8 h light: dark cycle. *A. pisum* (pea aphid) and *M. persicae* (peach potato aphid) were reared on broad bean (*Vicia faba*) and Chinese cabbage (*Brassica rapa*), respectively, and both colonies were maintained at 22 **°**C with a 16 h:8 h, light:dark photoperiod.

### 2.7 *Mamestra brassicae* injection assays

Newly eclosed 5th stadium *M. brassicae* larvae (average wt. 60 mg) were anaesthetised with CO_2_ and injected behind the head capsule with 5 μL of protein‐containing solutions in SPB. Controls were injected with 5 μL SPB. For each protein, five doses (ranging from 2.5 to 40 μg per larva) and ten larvae per dose were injected, and survival was monitored daily for a minimum of 4 days.

### 2.8 Aphid feeding and choice assays

Oral toxicity to *A. pisum* and *M. persicae* was determined using cylindrical feeding chambers overlain with parafilm sandwiches that contained proteins dissolved in liquid artificial diet.^27^ Stock protein solutions in SPB were added to sterile diet such that 100 μL diet contained 25 μL protein solution. Control diets contained an equivalent volume of SPB to the protein treatments. One‐day‐old nymphs, selected from adults maintained on artificial diet for 24 h, were placed on 25 μL artificial diet (15 nymphs per dose). Diets were replaced every 2 days and survival recorded daily. Preliminary assays enabled determination of appropriate ranges of protein concentrations to allow derivation of median lethal (LC_50_) concentrations.

Choice assays were performed in a similar way to oral toxicity bioassays except that 25 μL diet containing 0.6 mg mL^−1^ of a given test protein was placed alongside 25 μL control diet between two layers of parafilm on a feeding chamber such that the diets did not mix. Ovalbumin was used as a control protein treatment. Twenty Day (D)1 nymphs were placed between two diets (three replicates per choice test) and the number of aphids feeding on each diet was recorded after 24 and 48 h.

### 2.9 Aphid (*A. pisum*) topical assays

A topical protein delivery method was developed based upon published procedures,^28^ except that adult pea aphids were temporarily immobilised using CO_2_ and proteins were re‐suspended in water containing 0.1% (v/v) Breakthru. Breakthru is a nonionic agricultural adjuvant that aids spreading and allows rapid coverage of hydrophobic surfaces such as the insect cuticle. Anaesthetised aphids were individually placed in ventral contact with a 0.5 μL droplet of protein solution, left for 12 min, and then placed in feeding chambers. Preliminary experiments identified suitable protein concentrations and the appropriate adjuvant. Three biological replicates (15 aphids per replicate) were conducted for each treatment and dose; survival was recorded 24 h post‐treatment. Western analysis was performed on protein extracts of whole aphids that had been topically treated and then fed on control diet for 2 h and 18 h as follows; aphids (ten per sample) were washed to remove nonpenetrating proteins by immersion in 20% EtOH, ground with a micropestle in the presence of 100 μL 5× SDS‐sample buffer [containing 10% (v/v) β‐mercaptoethanol], boiled for 10 min, and centrifuged before loading 30 μL per lane on gel.

### 2.10 Fluorescent microscopy

Pea aphids were fed on FITC‐labelled proteins in diet at sublethal equimolar concentrations (HxTx‐Hv1h 0.38 mg mL^−1^, GNA 0.75 mg mL^−1^, HxTx‐Hv1h/GNA 1 mg mL^−1^) for 24 h and then transferred to control diet for a chase period of up to 24 h. Controls were fed on diet containing FITC and 0.1 mg mL^−1^ propidium iodide (PI) to enable visualisation of the gut. A subset of aphids (6 per treatment) fed on labelled proteins were retained as individuals in feeding chambers to allow emerging nymphs to be visualised. For contact assays, aphids were ‘dipped’ in labelled protein solutions as described previously and placed on control diets for ≤18 h. Before visualisation, aphids were washed by immersion in 20% EtOH. Aphids (9–12 per treatment and time point) were visualised using a fluorescent microscope (MC165; Leica, Wetzlar, Germany) under FITC filter (absorbance 494 nm; emission 521 nm) and images captured in openlab.

### 2.11 Statistical analysis

Statistical analysis was carried out using prism (GraphPad, San Diego, CA, USA) or Excel (Microsoft, Redmond, CA, USA) software. Survival data were analysed using Kaplan–Meier survival analysis. Median lethal doses or concentrations were calculated based upon predicted masses for the protein products by plotting log‐transformed data and nonlinear regression, constrained for control survival where necessary. Multiple Student's *t*‐tests were performed on contact assays and choice assay results were analysed using Chi‐square tests for significant differences between single values.

## 3 RESULTS

### 3.1 Recombinant protein production in the yeast *P. pastoris*

Synthetic genes encoding HxTx‐Hv1h and HxTx‐Hv1h/GNA were cloned in‐frame with the yeast alpha factor in the expression vector pGAPZαB by PCR amplification, followed by restriction digestion and ligation. A fusion protein was generated by fusing the HxTx‐Hv1h peptide to the N‐terminus of GNA via an 8‐aa residue, (Gly‐Gly‐Gly‐Gly‐Ser‐Ala‐Ala‐ Ala) linker region as depicted in Fig. 1(a). Both constructs contain a six‐residue histidine tag at the C‐terminus to enable affinity purification, and two additional N‐terminal residues; Gly and Ser (GS), reported to enhance the expression levels of HxTx‐Hv1h in the yeast *P. pastoris*.^29^ Constructs were cloned into *E. coli* and sequenced plasmid DNAs were linearised and transformed into competent *P. pastoris* cells. Small‐scale screening by Western blotting for protein expression enabled the selection of clones for bench‐top fermentation to produce sufficient quantities of proteins for insect bioassays. *P. pastoris* cells were grown in a laboratory fermenter and all proteins expressed at levels of >30 mg L^−1^ in culture supernatants. Proteins were purified from clarified supernatants by nickel‐affinity chromatography, followed by dialysis and freeze‐drying.

As shown in Fig. 1(b), purified HxTx‐Hv1h separates as two protein products of ≈17 kDa on SDS‐PAGE gels, which is more than double the predicted mass of 6.29 kDa. Immunoreactivity with anti‐His antibodies [Fig. 1(c)] provides evidence that both proteins represent recombinant toxin. LC–MS analysis further confirmed that both proteins are full length and have an identical 57 aa residue sequence (Appendix S1). The higher than predicted mass for recombinant HxTx‐Hv1h protein products on SDS‐PAGE gels is thought to be a consequence of incomplete denaturation by reducing agents (dithiothreitol and beta‐mercaptoethanol) and hyperglycosylation. No difference in the migration of proteins treated with the denaturing agents TCEP or thioglycolyic acid was observed on SDS‐PAGE gels (results not shown). Hyperglycosylation is commonly observed during expression of recombinant proteins in *P. pastoris* and is evidenced by periodic acid Schiff glycoprotein staining of recombinant proteins (Online Resource 1d). Purified HxTx‐Hv1h/GNA stained as a single protein of ≈20 kDa on SDS‐PAGE gels [*c*. 2 kDa higher than its predicted molecular mass; Fig. 1(b)] and reacted positively with anti‐GNA and anti‐His antibodies [Fig. 1(c)]. LC–MS analysis confirmed the presence of full‐length sequence (Online Resource 1) and that both proteins contain an additional alanine as a consequence of gene insertion via a *Pst*I restriction site in the pGAPZαB vector. As reportedpreviously,^21^ recombinant GNA which contains a C‐terminal histidine tag runs at ≈14 kDa on SDS‐PAGE gel [Fig. 1(b)], close to its predicted molecular mass of 12.8 kDa. Separation of protein samples by native gel electrophoresis is shown in Fig. 1(d). Whilst native electrophoresis does not allow for accurate molecular mass determination, it can be seen that HxTx‐Hv1h migrates further, as compared to GNA or HxTx‐Hv1h/GNA; this is observed under both nondenatured and – even more so –denatured conditions, and is indicative of a lower mass than that observed on SDS‐PAGE gels. As for SDS‐PAGE, two HxTx‐Hv1h proteins in the nondenatured sample in Fig. 1(d) were observed and may be indicative not only of a degree of dimerization, but also differences in disulfide bridge formation. The presence of clearly stained bands for all three proteins under nondenaturing conditions contrasts the protein smears observed when samples are denatured and provides evidence for disulfide bridge formation. The functionality of the GNA component of the fusion protein was confirmed by *in vitro* agglutination assays (results not shown).

### 3.2 Biological activity of recombinant proteins

#### 3.2.1 *Injection toxicity or recombinant proteins to lepidopteran larvae (*M. brassicae*)*

The biological activity of HxTx‐Hv1h and HxTx‐Hv1h/GNA was evaluated by injection of 2.5–40 μg of purified recombinant proteins into 5th stadium *M. brassicae* larvae (mean wt. 60 mg). Larvae injected with higher doses of toxin (>5 μg) or fusion protein (> 30 μg) displayed a paralytic phenotype 10–20 min post injection and dose‐dependent effects on larval mortality were observed predominantly within the first 48 h following treatment. Similar D4 median lethal doses of 16.29 nmols g^−1^ larvae [confidence interval (CI) perfect fit] and 13.80 nmols g^−1^ (CI 12.0–15.80 nmols) were derived for HxTx‐Hv1h and HxTx‐Hv1h/GNA, respectively. Paralysis and larval mortality verify that the recombinant toxin is functional when expressed alone or when fused to the N‐terminus of GNA.

#### 3.2.2 Oral toxicity of recombinant proteins to aphids

**Figure 2:**
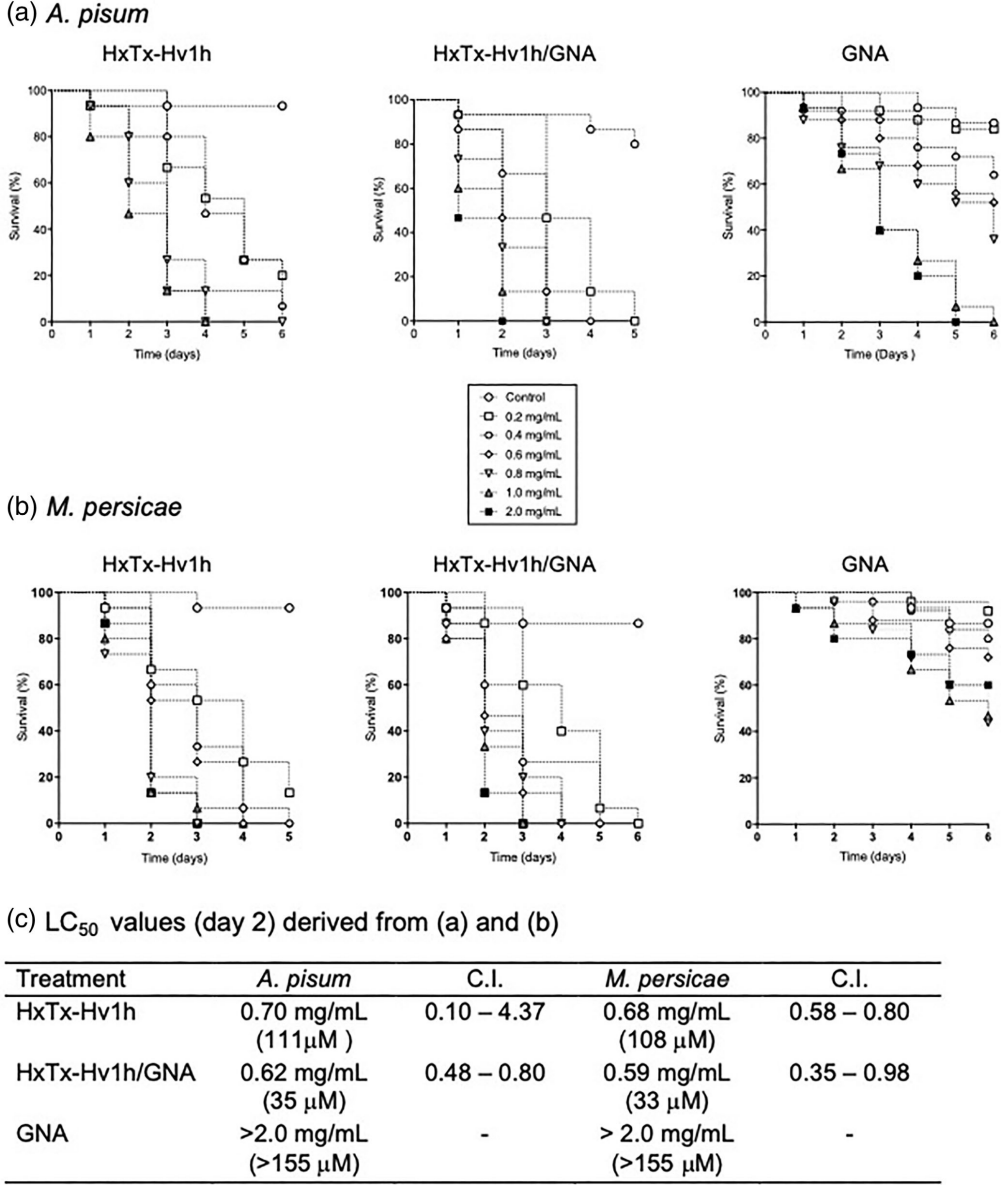
Survival of (a) *A. pisum* and (b) *M. persicae* fed on diets containing different concentrations of HxTx‐Hv1h, HxTxHv1h/GNA or GNA. (c) Day 2 LC_50_ values (mg mL^−1^; derived from bioassay data in (a) and (b). CI, confidence intervals; numbers in brackets, LC_50_ values in μm.

Oral toxicity was determined by feeding *A. pisum* or *M. persicae* nymphs with artificial diets containing a range of concentrations (0.2–2.0 mg mL^−1^) of purified HxTx‐Hv1h, HxTx‐Hv1h/GNA or GNA. As shown in Fig. 2, dose‐dependent reductions in the survival of aphids fed on protein‐containing diets were observed in all assays whereas control (no added protein diet) survival was >85%. HxTx‐Hv1h alone was toxic towards both species with 100% mortality observed after 4 days of feeding on diets containing >0.6 mg mL^−1^ of protein and comparable LC_50_ (D2) values of 111 μm (0.70 mg mL^−1^) and 108 μm (0.68 mg mL^−1^) were derived for pea and peach potato aphids, respectively. The fusion protein also showed acute oral toxicity to both species; however, D2 LC_50_ values were >3‐fold lower, as compared to HxTx‐Hv1h, at 35 μm (0.62 mg mL^−1^) and 33 μm (0.59 mg mL^−1^) for pea and peach potato aphids, respectively. Feeding on GNA alone caused a significant reduction in the survival of *A. pisum* at dietary concentrations of ≥0.6 mg mL^−1^ (i.e. > 46 μm) (Log Rank Mantel‐Cox; *P* < 0.05) whereas no significant differences in *M. persicae* survival as compared to control‐fed aphids were observed for any of the GNA treatments. The much reduced oral efficacy of GNA, as compared to HxTx‐Hv1h and HxTx‐Hv1h/GNA, prevented derivation of a defined LC_50_ for *A. pisum* or *M. persicae*.

**Figure 3:**
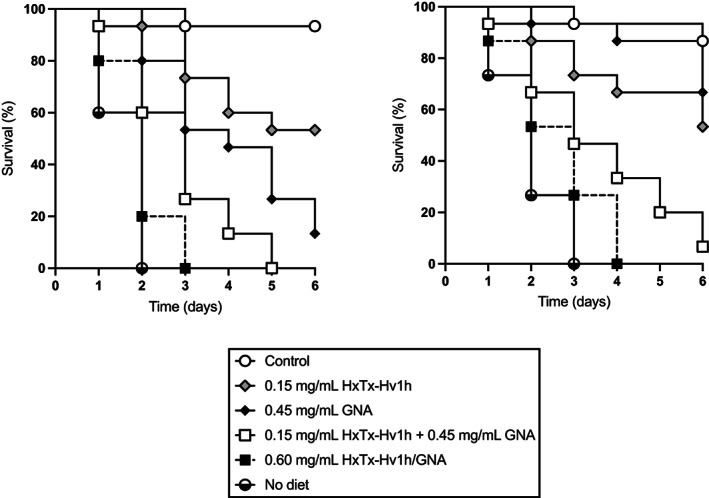
Survival of (a) *A. pisum* and (b) *M. persicae* fed on diets containing HxTx‐Hv1h, GNA, HxTx‐Hv1h/GNA, and an equivalent mixture of HxTx‐Hv1h and GNA.

In choice assays, both aphid species showed a preference for feeding on control (no added protein) diet over each of the recombinant proteins or peptide, whereas no preference for the control *versus* ovalbumin (control nontoxic protein) diets was observed (Online resource 2.) These results suggest that the observed mortality of aphids fed on protein‐containing diets (Figs 2 and 3) is at least, in part, a result of antifeedant effects.

Further aphid bioassays were conducted to verify that the enhanced efficacy of HxTx‐Hv1h/GNA was attributable to the direct action of the fusion protein rather than additive effects of feeding a combination of the toxin and GNA. As shown in Fig. 3, 100% mortality of *A. pisum* or *M. persicae* was observed after 3 or 4 days after feeding on diets containing 0.6 mg mL^−1^ fusion protein, respectively. By comparison, feeding on a combination of HxTx‐Hv1h (0.15 mg mL^−1^) and GNA (0.45 mg mL^−1^) resulted in 100% *A. pisum* and 93% *M. persicae* mortality after 5 days of feeding. Survival curves for fusion protein and the combination treatments were significantly different (*P* = 0.010 *A. pisum* and *P* = 0.016 *M. persicae*; Mantel–Cox, log‐rank test). Combining both toxin and GNA resulted in additive effects upon aphid survival; for example, after 3 days of feeding respective 27% and 47% pea aphid mortalities were observed for HxTx‐Hv1h and GNA treatments, as compared to 73% mortality for the treatment containing a mixture of toxin and GNA. That mortality was attributable to ingestion of the protein and not simply to antifeedant effects is indicated by the slower onset of full mortality as compared to the no diet control treatment.

**Figure 4:**
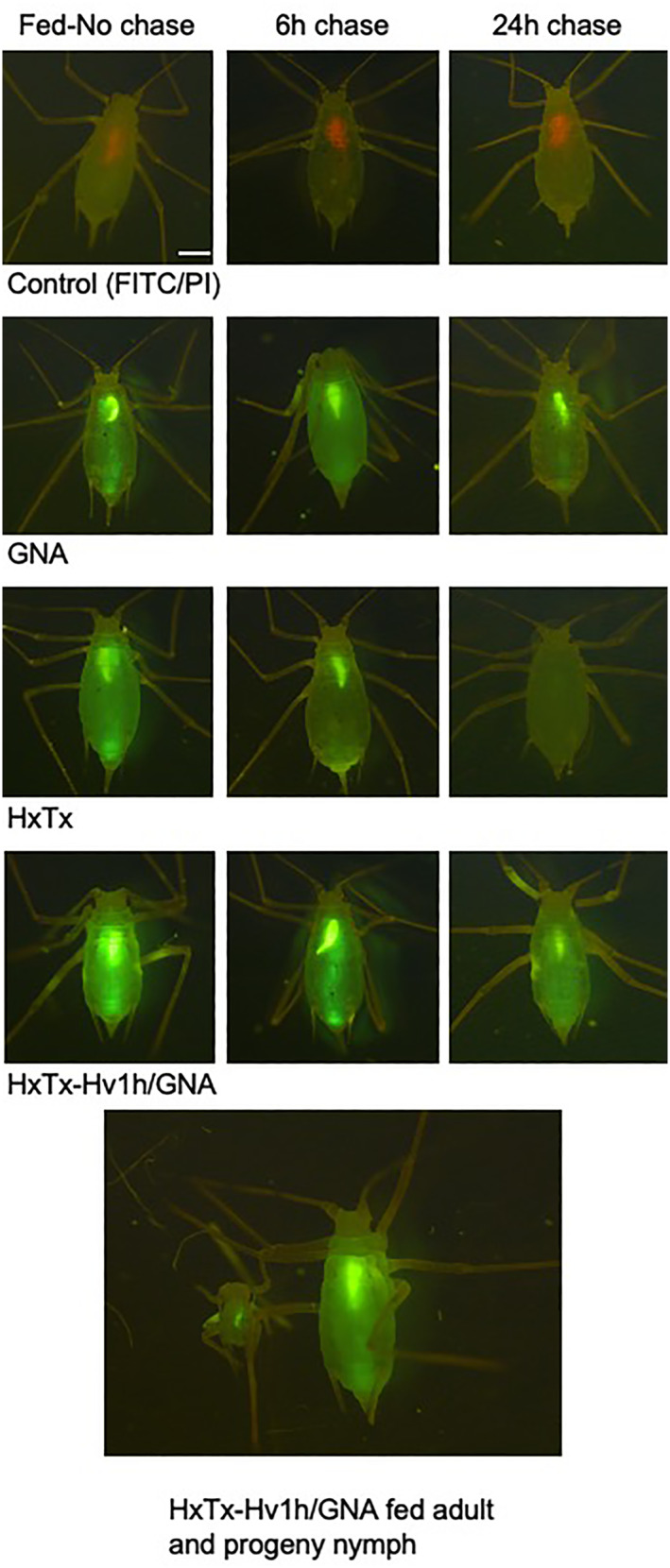
Composite of whole *A. pisum* fed on diets containing FITC‐labelled proteins or control‐FITC/PI for 24 h followed by chase feeding on control diets for 6 h and 24 h. Images were visualised with a fluorescent microscope under the FITC filter and captured in openlab Scale bar = 0.5 mm. Bottom frame shows an adult (fed on FITC‐fusion protein for 24 h, placed in a feeding chamber overnight) and emerged nymph.

Fluorescence imagery of whole *A. pisum* chase‐fed control diet after feeding on equimolar concentrations of FITC labelled toxin, fusion protein or GNA for 24 h is presented in Fig. 4. Visualisation of the foregut is evident in control PI fed aphids. Labelled proteins were readily detectable in aphids after 24 h of feeding and fluorescence (particularly in the gut region) persisted for a chase‐feed period of 6 h suggesting that all proteins were all able to bind to the gut epithelium. Fluorescence persisted in GNA and fusion protein‐fed aphids after 24 h of chase feeding but was notably absent in HxTx‐Hv1h‐fed aphids, suggesting comparatively weaker binding and more rapid clearance of the toxin. Transport of fusion protein across the aphid gut to the circulatory system is indicated by whole body fluorescence in adult aphids and fluorescence of the gut region in progeny derived from HxTx‐Hv1h/GNA fed adults. This was not observed for progeny derived from aphids fed HxTx‐Hv1h or GNA alone although sample numbers were limited (four to five nymphs per treatment) as very few aphids produced nymphs.

#### 3.2.3 *Contact toxicity in* A. pisum

**Figure 5:**
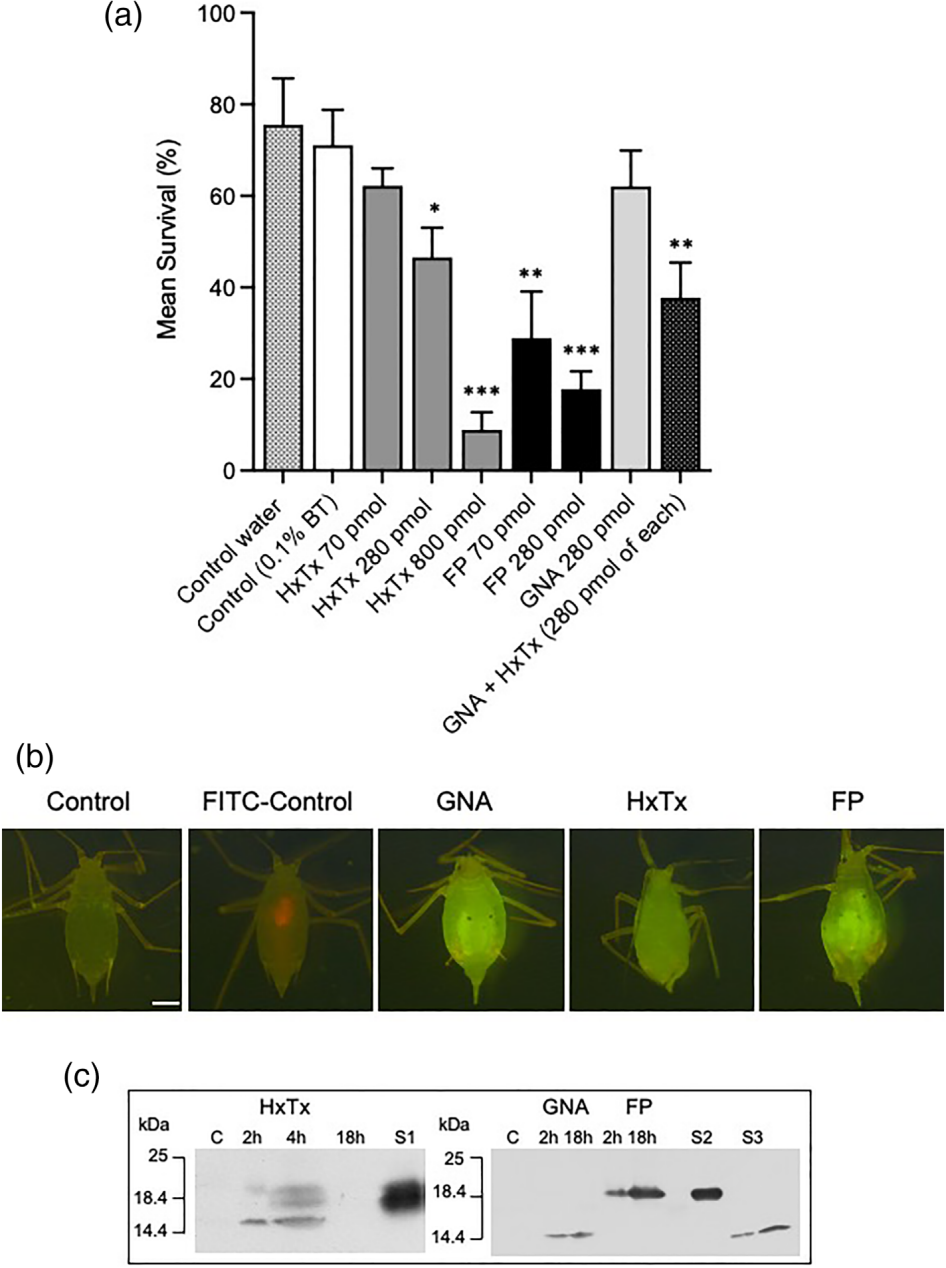
(a) Pea aphid survival 24 h postcontact exposure to water, water+ Breakthru (BT), HxTx‐Hv1h (HxTx), GNA, HxTx‐Hv1h/GNA (FP), or a mixture of GNA and toxin. All protein treatments contained BT. Bars depict standard error of the mean (three replicates of *n* = 15 per dose). Significant differences to control (+BT) (by Student's *t*‐tests): *, *P* < 0.05; **, *P* < 0.005; ***, *P* < 0.0005. (b) Representative images of aphids taken 18 h postcontact with droplets containing 70 pmol FITC‐labelled HxTx‐Hv1h (HxTx), GNA or HxTx‐Hv1h/GNA (FP). Aphids were fed on control diet and washed before imaging under FITC filter captured in openlab. Scale bar = 0.5 mm. (c) Western analysis (anti‐His antibodies) of whole aphid protein samples extracted 2 h and 18 h postcontact with droplets containing HxTx‐Hv1h, GNA or fusion protein. S1, HxTx‐Hv1h standard (250 ng); S2, fusion protein (200 ng); and S3, recombinant GNA (50 and 100 ng).

The efficacy of topically applied proteins was evaluated by placing pea aphids in ventral contact with droplets containing different concentrations of HxTx‐Hv1h, HxTx‐Hv1h/GNA or GNA [Fig. 5(a)]. Mean survival of aphids exposed to water only control was *c*. 80% as compared to 70% for Break‐thru alone (BT control) and 62% for GNA (280 pmol). Differences between water control and BT or GNA were not significant suggesting that neither Breakthru nor GNA had significantly detrimental effects upon aphid survival. Exposure to 70 pmol HxTxHv1h did not cause a significant reduction in survival as compared to the BT control treatment. By contrast, significant dose‐dependent reductions in aphid survival were observed for HxTx‐Hv1h (at 280 pmol and 800 pmol; *P* = 0.0142 and *P* = 0.0002, respectively) and fusion protein treatments (70 pmol and 280 pmol; *P* = 0.0046 and *P* = 0.0004, respectively) as compared to the BT control group. Significant differences between survival of aphids treated with fusion protein and toxin only were observed for both 70 pmol and 280 pmol treatments (respectively, *P* = 0.0061 and 0.0029, Student's *t*‐tests). Furthermore, a significant reduction in survival was observed between aphids exposed to droplets containing 280 pmol of fusion protein or a combination of 280 pmol each of GNA and HxTx‐Hv1h (*P* = 0.0158; Student's *t*‐test). These results provide evidence that fusion to GNA significantly enhances contact efficacy of HxTx‐Hv1h towards aphids.

Delivery of proteins across the cuticle following contact exposure to labelled proteins is shown in Fig. 5(b). After ‘dipping’ in protein solutions, aphids were fed on control diet for 18 h, washed and imaged. An absence of fluorescence in control‐treated aphids contrasts whole‐body fluorescence observed in GNA, HxTx‐Hv1h and fusion protein‐treated aphids, and provides evidence for protein delivery across the cuticle. The intensity of fluorescence appeared generally greater in fusion protein and GNA‐dipped aphids as compared to HxTx‐Hv1h. HxTx‐Hv1h was detected by Western analysis [Fig. 5(c)] of whole aphid protein extracts prepared 2 and 4 h postcontact exposure, but was not detectable in samples prepared 18 h post‐treatment. By contrast, GNA and HxTx‐Hv1h/GNA both were detected in immunoblotted extracts prepared 2 h and 18 h postcontact treatment. Whilst all samples were probed with anti‐His antibodies, it is possible that the histidine tag was cleaved from HxTx‐Hv1h‐treated aphids in the 18 h sample (preventing detection) but remained intact in GNA and fusion protein‐treated insects.

## 4 DISCUSSION

The HxTx‐Hv1h toxin is sold as a contact pesticide for foliar spray induced control of glasshouse sap‐sucking pests. The purpose of this study was to produce this venom neuropeptide and a fusion protein comprising HxTx‐Hv1h fused to the N‐terminus of the carrier protein GNA using yeast as an expression host, and subsequently to conduct a comparative assessment of insecticidal efficacy towards two species of aphids (*A. pisum* and *M. persicae*). Recombinant proteins were purified by nickel affinity chromatography from fermented *P. pastoris* cultures that were immunoreactive with anti‐His and/or anti‐GNA antibodies, and characterised as full‐length products by LC–MS. Analysis by native PAGE together with *M. brassicae* injection assays that demonstrate both toxin and fusion protein cause paralysis and larval mortality, provides indirect evidence of correct disulfide bridge formation in the expressed products. Further studies, such as comparing the injection toxicity of recombinant HxTx‐Hv1h with native toxin, would be required to elucidate whether or not disulfide connectivity is compromised in yeast‐expressed forms of the peptide.

When injected into *M. brassicae* larvae dose‐dependent mortality predominantly occurred 24–72 h postinjection, and similar respective LD_50_ values of 16.29 and 13.70 nmoles g^−1^ for HxTx‐Hv1h and HxTx‐Hv1h/GNA were derived. These values are comparable to injection LD_50_ values of 18 nmol g^−1^ *M. brassicae* larvae reported previously for both Hv1a and Hv1a/GNA.^22^ This contrasts with previous reports showing much higher potency of HxTx‐Hv1h (LD_50_ 38 pmol g^−1^) as compared to Hv1a (LD_50_ 87 pmol g^−1^) when injected into houseflies.^10^, ^11^ However, a cautionary approach should be taken when comparing efficacy across different insect species that show large variability in levels of susceptibility to venom‐derived neurotoxins. This variability is attributable to a multitude of factors including toxin derivation (synthetic, recombinant, or native), delivery method, the diverse nature and affinity for target channels across insect species, as well as *in vivo* stability against proteolytic degradation.

Whilst HxTx‐Hv1h injection toxicity was comparable to that reported for Hv1a, when fed to aphids HxTx‐Hv1h was found to be by far the more insecticidal of the two toxins, supporting previous data showing it to be the more potent toxin.^10^, ^11^ HxTx‐Hv1h was acutely toxic to aphids causing 100% mortality of *A. pisum* and *M. persicae* after 3–4 days of feeding on diets containing >0.6 mg mL^−1^ protein. HxTx‐Hv1h was similarly toxic to pea and peach potato aphids with LC_50_ (D2) values of 0.70 and 0.68 mg mL^−1^, respectively. By comparison, we previously found the hexatoxin, Hv1a, when produced as a recombinant protein, to be considerably less orally toxic to aphids with reported LC_50_ doses (D2) of 1.01 and 2.34 mg mL^−1^ for *A. pisum* and *M. persicae*, respectively.^30^ Greater oral toxicity of HxTx‐Hv1h observed herein as compared to previously published data for Hv1a, is in agreement with Sollod (2006)^11^ who reported HxTx‐Hv1h to be more efficacious than Hv1a by injection into houseflies. Both toxins are known to act as positive allosteric modulators of nAChRs and, at least for Hv1a, agonist binding assays together with electrophysiological studies have suggested this to be the most potent molecular effect of the toxin.^13^ As Hv1a and HxTx‐Hv1h are both orally toxic towards aphids, this suggests that at least some of the ingested toxin traverses the gut epithelium and is able to target nAChRs in the central nervous system. Fluorescence in the *A. pisum* gut and body cavity after feeding on diets containing FITC‐labelled toxin, and persistence in the gut region after chase feeding with control diets, indicates that HxTx‐Hv1h is able to bind and traverse the gut epithelium. The greater oral toxicity of HxTx‐Hv1h as compared to Hv1a may be attributable to a number of factors including: greater stability against proteolytic digestion in the aphid gut, differences in ability to bind to the gut and differential affinity for nAChRs in the CNS. Whilst not assessed here, further studies would be required to determine if comparable levels of toxin and fusion protein were ingested by the aphids. HxTx‐Hv1h as a hybrid toxin also may show enhanced oral activity owing to its ability to synergistically disrupt both Ca_v_ and K^+^ channels, whereas Hv1a activity is thought to be restricted to the inhibition of Ca_v_.^1^, ^4^, ^5^, ^31^ That both toxins are able to target sites within the peripheral nervous system also is a possibility.

As for HxTx‐Hv1h, the HxTx‐Hv1h/GNA fusion protein also was found to be similarly orally toxic to both aphid species. However, LC_50_ values for HxTx‐Hv1h/GNA were >3‐fold lower on a molar basis than those for HxTx‐Hv1h, demonstrating that fusion to GNA significantly enhances the oral efficacy of the toxin. Furthermore, the significant difference in mortality of both species when fed on fusion protein as compared to an equivalent mixture of toxin and GNA further suggests that enhanced efficacy is attributable to the ability of GNA to deliver linked toxin across the gut epithelium to its target sites of action in the CNS. This is further supported by the observed enhanced persistence of fluorescence in gut and body cavities of fusion protein‐ and GNA‐fed pea aphids in chase feed experiments, and is similar to that reported previously for recombinant venom peptide δ‐amaurobitoxin PI1a and PI1a/GNA.^22^ A number of studies likewise have reported that the efficacy of a toxin is enhanced when it is genetically fused to plant lectins. For example, the galactose‐binding component of ricin and mannose‐binding lectins from garlic and onion have been fused with Cry1Ac *Bacillus thuringiensis (Bt)* toxins and ω‐hexatoxin Hv1a, expressed *in planta* and shown to enhance insecticidal activity, as compared to the toxins alone, towards hemipteran and lepidopteran pests.^32^, ^33^, ^34^, ^35^

We report for the first time the insecticidal efficacy of topically applied recombinant fusion proteins towards pea aphids in the light of the current use of HxTx‐Hv1h as a contact foliar spray that targets sap‐sucking pests. Significant dose‐dependent reductions in survival were observed after ‘dipping’ aphids in protein solutions (in the presence of Breakthru) for toxin and fusion protein, but not for GNA alone. Surprisingly, although reductions in survival were comparable for HxTx‐Hv1h and HxTx‐Hv1h/GNA on a total protein basis, molar comparisons suggest (as observed in feeding assays) that fusion to GNA potentiates contact efficacy. Again, as observed in feeding assays, that enhanced activity is attributable to the fusion of HxTx‐Hv1h to GNA rather than additive effects of the GNA protein and toxin was confirmed by the significantly greater reduction in survival of fusion protein‐treated aphids as compared to those exposed to an equivalent mixture of GNA and toxin. Fluorescence microscopy and Western blotting of whole aphids allowed visualisation of internalised proteins after contact treatments. Enhanced intensity of fluorescence was observed 18 h post‐treatment for GNA‐ and HxTx‐Hv1h/GNA‐ as compared to HxTx‐Hv1h‐treated aphids. Immunoblot analysis of aphid protein extracts also provided evidence for enhanced persistence of fusion protein and GNA in contact‐treated aphids. These results suggest that enhanced contact efficacy may be attributable to binding of GNA to glycoproteins within the aphid. Further studies would require the use of an anti‐HxTx‐Hv1h antibody to comparatively assess the levels of toxin persisting postcontact exposure to either toxin or fusion protein. We previously reported the ability of GNA and Hv1a/GNA to bind to the central nerve chord of lepidopteran (*M. brassicae*) larvae and we suggest that a similar mode of toxin delivery to the CNS of aphids may occur following contact exposure.^19^ In addition to causing aphid mortality directly (either by ingestion or contact), antifeedant effects also were observed that may offer additional crop protection benefits via indirect action as a feeding deterrent.

## 5 CONCLUSIONS

We have demonstrated that a recombinant HxTx‐Hv1h venom‐derived neuropeptide has oral and contact activity against aphid pests. However, whilst HxTx‐Hv1h alone is toxic, we demonstrate that fusion to GNA potentiates both oral and contact efficacy towards aphids in laboratory assays. By analogy, the commercial HxTx‐Hv1h product is recommended for use in combination with a low dose of *BtK* (*Bt* var. *kurstaki*) that due to its ability to form pores in the midgut of certain insect pests, enhances delivery of the HxTx‐Hv1h toxin to the CNS. Thus, a fusion protein‐based approach may offer an opportunity to significantly enhance oral and contact efficacy of toxins, such as HxTx‐Hv1h, towards pests including those that are resistant to the effects of *Bt* toxins.

## AUTHOR CONTRIBUTIONS

PP and CW created expression constructs and carried out preliminary expression studies; FS produced recombinant proteins for testing and FS and JR conducted aphid assays; APB carried out mass spectrophotometry; and EF wrote the manuscript.

## Conflict of interest

The authors declare they have no conflict of interest.

## Ethical APPROVAL

This article does not contain any studies with human participants or animals.

## Supporting information


**Appendix S1.** Supporting information.

## Data Availability

Data available on request from the authors The data that support the findings of this study are available from the corresponding author upon reasonable request.

## References

[1] Pineda SS , Chaumeil PA , Kunert A , Kaas Q , Thang MWC , Le L *et al*., ArachnoServer 3.0: an online resource for automated discovery, analysis and annotation of spider toxins. Bioinformatics 34:1074–1076 (2018). 29069336 10.1093/bioinformatics/btx661

[2] Pallaghy PK , Nielsen KJ , Craik DJ and Norton RS , A common structural motif incorporating a cystine knot and a triple‐stranded β‐sheet in toxic and inhibitory polypeptides. Protein Sci 3:1833–1839 (1994). 7849598 10.1002/pro.5560031022 PMC2142598

[3] Craik DJ , Daly NL and Waine C , The cystine knot motif in toxins and implications for drug design. Toxicon 39:43–60 (2001). 10936622 10.1016/s0041-0101(00)00160-4

[4] Herzig V and King G , The cystine knot is responsible for the exceptional stability of the insecticidal spider toxin x‐hexatoxin‐Hv1a. Toxins 7:4366–4380 (2015). 26516914 10.3390/toxins7104366 PMC4626739

[5] Herzig V , de Araujo AD , Greenwood KP , Chin YK , Windley MJ , Chong Y *et al*., Evaluation of chemical strategies for improving the stability and oral toxicity of insecticidal peptides. Biomedicine 6:90–105 (2018). 10.3390/biomedicines6030090 PMC6164231 30154370

[6] Atkinson RK , Howden MEH , Tyler MI and Vonarx EJ , Insecticidal Toxins Derived from Funnel Web Spider (Atrax or Hadronyche) Spiders. US Patent No 5763568, 1998.

[7] Atkinson RK , Vonarx EJ and Howden MEH , Effects of whole venom and venom fractions from several Australian spiders, including Atrax (*Hadronyche*) species, when injected into insects. Comp Biochem Physiol 114C:113±117 (1996).

[8] Fletcher JI , Smith R , O'Donoghue SI , Nilges M , Connor M , Howden ME *et al*., The structure of a novel insecticidal neurotoxin, v‐atracotoxin‐HV1, from the venom of an Australian funnel web spider. Nat Struct Biol 4:559–566 (1997). 9228949 10.1038/nsb0797-559

[9] Nakasu EY , Williamson SM , Edwards MG , Fitches EC , Gatehouse JA , Wright GA *et al*., Novel biopesticide based on a spider venom peptide shows no adverse effects on honeybees. Proc Biol Sci 281:20140619 (2014). 24898372 10.1098/rspb.2014.0619 PMC4071547

[10] Tedford HW , Gilles N , Ménez A , Doering CJ , Zamponi GW and King GF , Scanning mutagenesis of omega‐atracotoxin‐Hv1a reveals a spatially restricted epitope that confers selective activity against insect calcium channels. J Biol Chem 279:44133–44140 (2004). 15308644 10.1074/jbc.M404006200

[11] Sollod BL , From Venoms to Insecticides: Exploring the Structure, Function and Evolution of Peptide Toxins Found in the Venom of Australian Funnel‐Web Spiders. *PhD Thesis*. University of Connecticut, ProQuest Dissertations Publishing, 3205761 (2006).

[12] Chong Y , Hayes JL , Sollod BL , Wen S , Hains PG , Hodgson WC *et al*., The omega‐atrac otoxins: selective blockers of insect M‐LVA and HVA calcium channels. Biochem Pharmacol 74:623–638 (2007). 17610847 10.1016/j.bcp.2007.05.017

[13] Chambers C , Cutler P , Huang Y‐H , Goodchild JA , Blythe J , Wang CK *et al*., Insecticidal spider toxins are high affinity positive allosteric modulators of the nicotinic acetylcholine receptor. FEBS Lett 593:1336–1350 (2019). 31102259 10.1002/1873-3468.13435

[14] Bass C , Puinean AM , Zimmer CT , Denholm I , Field LM , Foster SP *et al*., The evolution of insecticide resistance in the peach potato aphid, *Myzus persicae* . Insect Biochem Mol Biol 51:41–51 (2014). 24855024 10.1016/j.ibmb.2014.05.003

[15] Jones AK , Raymond‐Delpech VR , Thany SH , Gauthier M and Sattelle DB , The nicotinic acetylcholine receptor gene family of the honey bee, *Apis mellifera* . Genome Res 16:1422–1430 (2006). 10.1101/gr.4549206 PMC1626644 17065616

[16] Saez NJ and Herzig V , Versatile spider venom peptides and their medical and agricultural applications. Toxicon 158:109–126 (2019). 30543821 10.1016/j.toxicon.2018.11.298

[17] Fitches E , Edwards MG , Mee C , Grishin E , Gatehouse AMR , Edwards JP *et al*., Fusion proteins containing insect‐specific toxins as pest control agents: snowdrop lectin delivers fused insecticidal spider venom toxin to insect haemolymph following oral ingestion. J Insect Phys 50:61–71 (2004). 10.1016/j.jinsphys.2003.09.010 15037094

[18] Fitches EC , Bell HA , Powell ME , Back E , Sargiotti C , Weaver RJ *et al*., Insecticidal activity of scorpion toxin (ButaIT) and snowdrop lectin (GNA) containing fusion proteins towards pest species of different orders. Pest Manage Sci 66:74–84 (2010). 10.1002/ps.1833 19728320

[19] Fitches EC , Pyati PS , King GF and Gatehouse JA , Fusion to snowdrop lectin dramatically enhances the oral activity of the insecticidal peptide ω‐hexatoxin‐Hv1a by mediating its delivery to the central nervous system. PLoS One 7:e39389 (2012). 22761779 10.1371/journal.pone.0039389 PMC3382250

[20] Trung NP , Fitches EC and Gatehouse JA , A fusion protein containing a lepidopteran‐specific toxin from the South Indian red scorpion (*Mesobuthus tamulus*) and snowdrop lectin shows oral toxicity to target insects. BMC Biotechnol 6:18 (2006). 16542451 10.1186/1472-6750-6-18 PMC1459149

[21] Yang S , Pyati P , Fitches E and Gatehouse JA , A recombinant fusion protein containing a spider toxin specific for the insect voltage‐ gated sodium ion channel shows oral toxicity towards insects of different orders. Insect Biochem Mol Biol 47:1–11 (2014). 24486516 10.1016/j.ibmb.2014.01.007 PMC4024200

[22] Powell ME , Bradish HM , Ca M , Makinson R , Brown AP , Gatehouse JA *et al*., Demonstrating the potential of a novel spider venom‐based biopesticide for target‐specific control of the small hive beetle, a serious pest of the European honeybee. J Pest Sci 93:391–402 (2019). 10.1007/s10340-019-01143-3 PMC6957549 31997983

[23] Bonning BC , Pal N , Li S , Wang Z , Sivakumar S , Dixon PM *et al*., Toxin delivery by the coat protein of an aphid‐vectored plant virus provides plant resistance to aphids. Nat Biotechnol 32:102–105 (2014). 24316580 10.1038/nbt.2753

[24] Cloyd RA and Herrick NJ , Effects of pesticides on the survival of rove beetle (Coleoptera: Staphylinidea) and insidious flower Bug (Hemiptera: Anthocoridae) adults. J Econ Entom 111:78–88 (2018). 29202189 10.1093/jee/tox280

[25] Fanning PD , Van Woerkom A , Wise JC and Isaacs R , Assessment of a commercial spider venom peptide against spotted‐wing Drosophila and interaction with adjuvants. JPest Sci 91:1279–1290 (2018).

[26] Hubbard CB and Gerry AC , Evaluation of spider venom toxin—based insecticide to control house flies. Arthropod Manag Tests 44:1–2 (2019).

[27] Prosser WA and Douglas AE , A test of the hypotheses that nitrogen is upgraded and recycled in an aphid (*Acyrthosiphon pisum*) symbiosis. J Insect Physiol 38:93–99 (1992).

[28] Niu J , Yang W‐J , Tian Y , Fan J‐Y , Ye C , Shang F *et al*., Topical dsRNA delivery induces gene silencing and mortality in the pea aphid. Pest Manage Sci 75:2873–2881 (2019). 10.1002/ps.5457 31038279

[29] Kennedy RM , Tedford W , Hendrickson C , Venable R , Foune C , Mcintyre J *et al*., Toxic Peptide Production, Peptide Expression in Plants and Combinations of Cysteine Rich Peptides. PCT Pub. No.WO 2013/134734 A2. Vestaron Corp, WIPO (2013).

[30] Bell J , Sukiran NA , Walsh S and Fitches EC , The insecticidal activity of recombinant nemertide toxin α‐1 from *Lineus longissimus* towards pests and beneficial species. Toxicon 79‐86 (2021). 10.1016/j.toxicon.2021.04.003 33852905

[31] Windley MJ , Herzig V , Dziemborowicz SA , Hardy MC , King GF and Nicholson GM , Spider‐venom peptides as bioinsecticides. Toxins 4:191–227 (2012). 22741062 10.3390/toxins4030191 PMC3381931

[32] Mehlo LM , Gahakwa D , Trung Nghia P , Thi Loc N , Capell T , Gatehouse JA *et al*., An alternative strategy for sustainable pest resistance in genetically enhanced crops. Proc Natl Acad Sci 102:7812–7816 (2005). 15908504 10.1073/pnas.0502871102 PMC1142385

[33] Boddupally D , Tamirisa S , Sivakrishna RG , Dashavantha RV and Khareedu VR , Expression of hybrid fusion protein (Cry1Ac:ASAL) in transgenic rice plants imparts resistance against multiple insect pests. Sci Rep 8:8458 (2018). 29855556 10.1038/s41598-018-26881-9 PMC5981619

[34] Javaid S , Naz S , Amin I , Jander G , Ul‐Haq Z and Mansoor S , Computational and biological characterization of fusion proteins of two insecticidal proteins for control of insect pests. Sci Rep 8:4837 (2018). 29556063 10.1038/s41598-018-23138-3 PMC5859112

[35] Rauf I , Javaid S , Naqvi RZ , Mustafa T , Amin I , Mukhtar Z *et al*., *In‐planta* expression of insecticidal proteins provides protection against lepidopteran insects. Sci Rep 9:6745 (2019). 31043622 10.1038/s41598-019-41833-7 PMC6494996

