# Supplementary figures and images for: Enhancing the oral and topical insecticidal efficacy of a commercialized spider venom peptide biopesticide via fusion to the carrier snowdrop lectin (Galanthus nivalis agglutinin)
Source: Pest Manag Sci. 2022 Oct 10;79(1):284–94. doi: 10.1002/ps.7198 (PMC10091797; doi:10.1002/ps.7198)

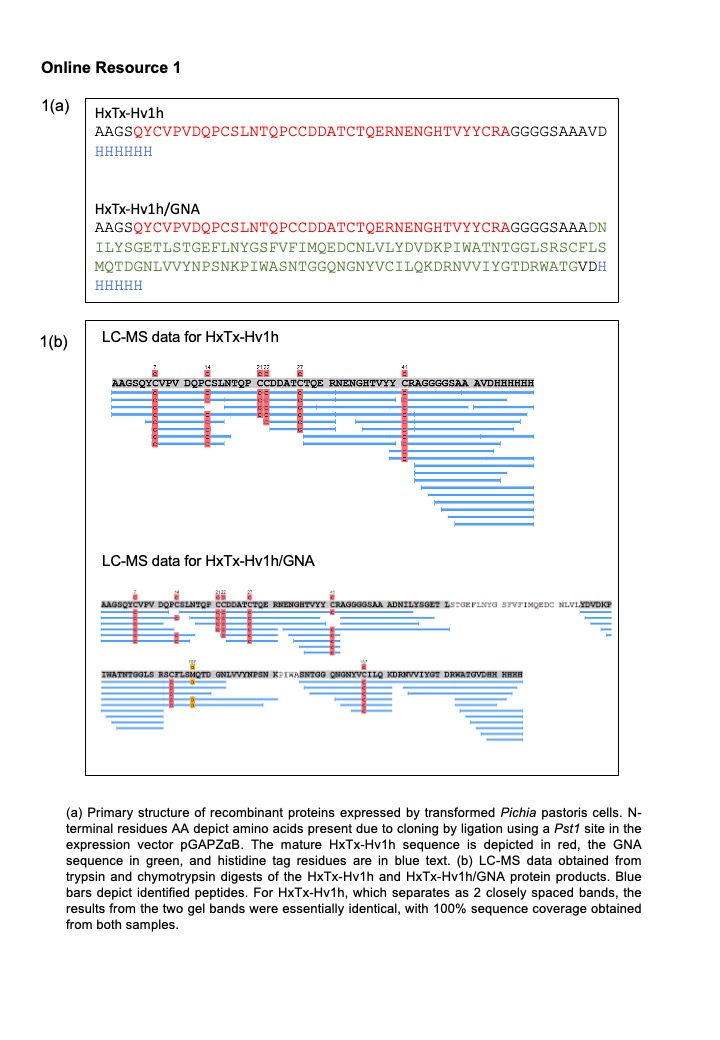

Supplement: Supplementary file 1 — Appendix S1. Supporting information. [file PS-79-284-s001.zip › Onlineres1a&amp;b.jpeg]

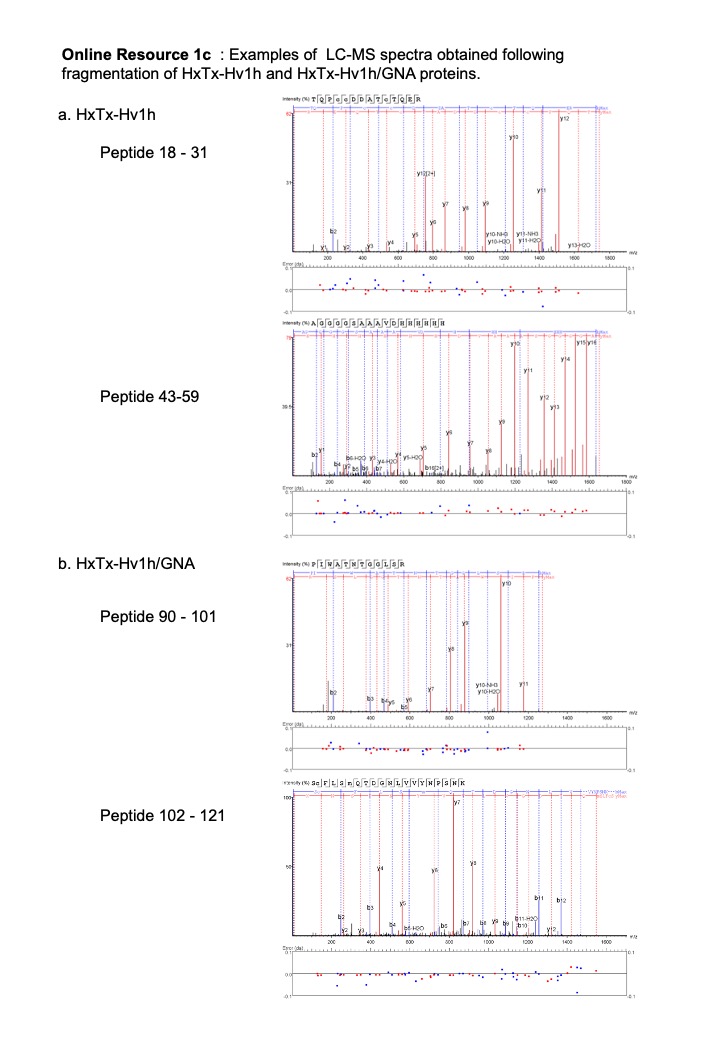

Supplement: Supplementary file 1 — Appendix S1. Supporting information. [file PS-79-284-s001.zip › OnlineRes1c.jpeg]

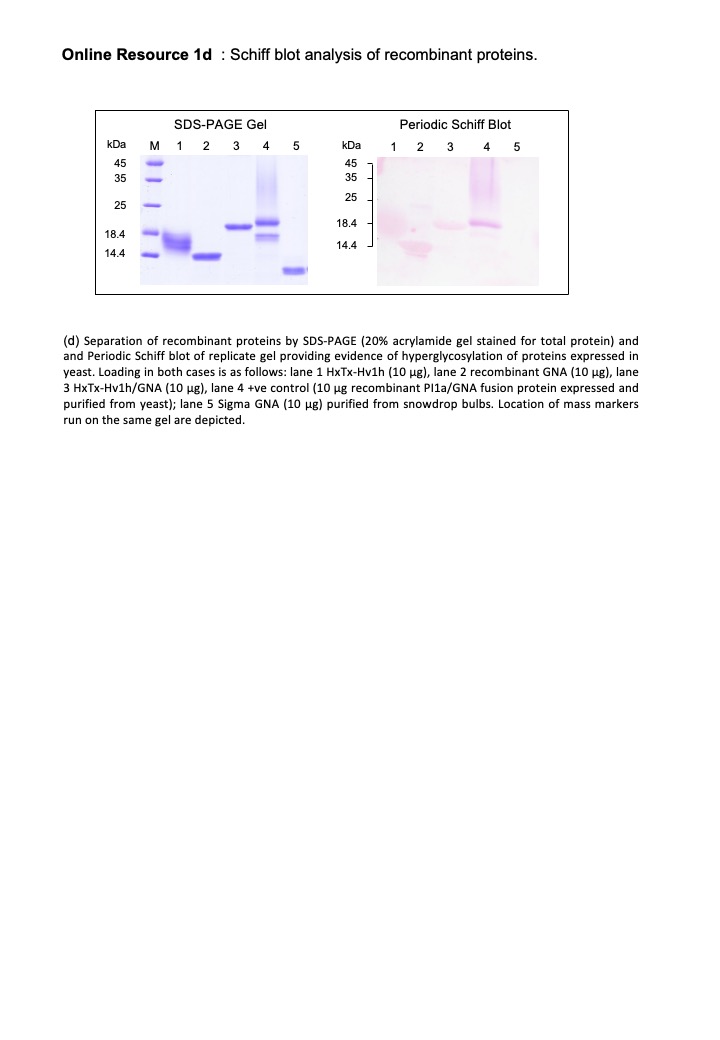

Supplement: Supplementary file 1 — Appendix S1. Supporting information. [file PS-79-284-s001.zip › OnlineRes1d.jpeg]

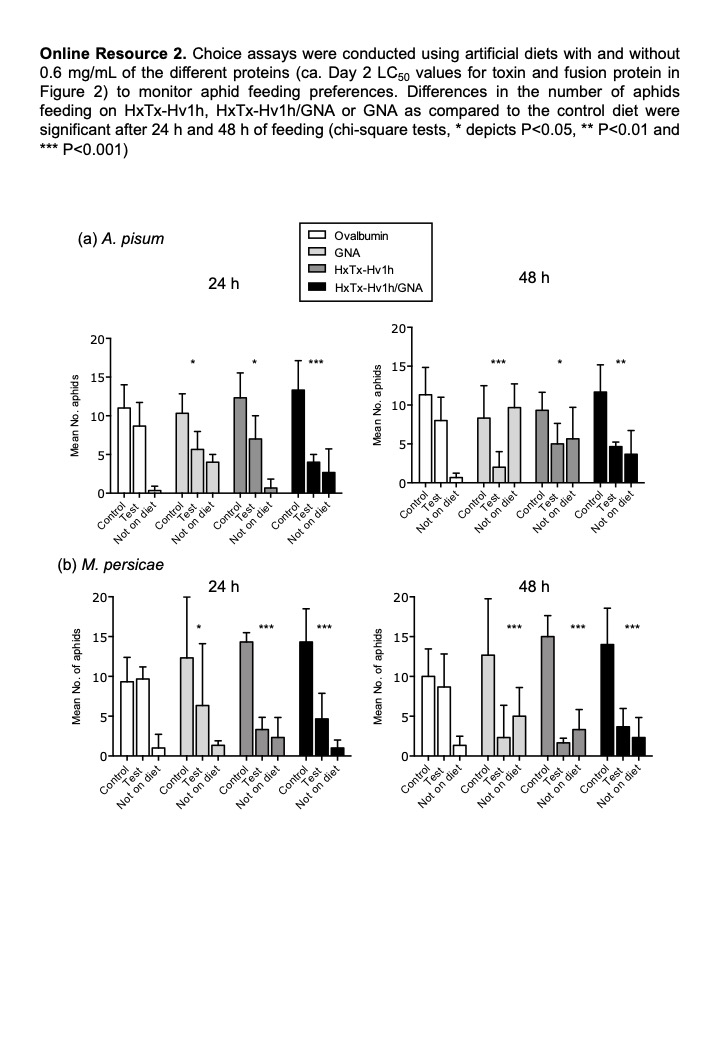

Supplement: Supplementary file 1 — Appendix S1. Supporting information. [file PS-79-284-s001.zip › Onlineresource2.jpeg]
